# Enhancement of Cellular Antioxidant-Defence Preserves Diastolic Dysfunction via Regulation of Both Diastolic Zn^2+^ and Ca^2+^ and Prevention of RyR2-Leak in Hyperglycemic Cardiomyocytes

**DOI:** 10.1155/2014/290381

**Published:** 2014-02-13

**Authors:** Erkan Tuncay, Esma N. Okatan, Aysegul Toy, Belma Turan

**Affiliations:** Department of Biophysics, Faculty of Medicine, Ankara University, Ankara 06100, Turkey

## Abstract

We examined whether cellular antioxidant-defence enhancement preserves diastolic dysfunction via regulation of both diastolic intracellular free Zn^2+^ and Ca^2+^ levels ([Zn^2+^]_*i*_ and [Ca^2+^]_*i*_) levels *N*-acetyl cysteine (NAC) treatment (4 weeks) of diabetic rats preserved altered cellular redox state and also prevented diabetes-induced tissue damage and diastolic dysfunction with marked normalizations in the resting [Zn^2+^]_*i*_ and [Ca^2+^]_*i*_. The kinetic parameters of transient changes in Zn^2+^ and Ca^2+^ under electrical stimulation and the spatiotemporal properties of Zn^2+^ and Ca^2+^ sparks in resting cells are found to be normal in the treated diabetic group. Biochemical analysis demonstrated that the NAC treatment also antagonized hyperphosphorylation of cardiac ryanodine receptors (RyR2) and significantly restored depleted protein levels of both RyR2 and calstabin2. Incubation of cardiomyocytes with 10 µM ZnCl_2_ exerted hyperphosphorylation in RyR2 as well as higher phosphorphorylations in both PKA and CaMKII in a concentration-dependent manner, similar to hyperglycemia. Our present data also showed that a subcellular oxidative stress marker, NF-**κ**B, can be activated if the cells are exposed directly to Zn^2+^. We thus for the first time report that an enhancement of antioxidant defence in diabetics via directly targeting heart seems to prevent diastolic dysfunction due to modulation of RyR2 macromolecular-complex thereby leading to normalized [Ca^2+^]_*i*_ and [Zn^2+^]_*i*_ in cardiomyocytes.

## 1. Introduction

Diabetic cardiomyopathy was first recognized by Rubler et al. [1] in diabetic humans with congestive heart failure without any evidence of coronary atherosclerosis. It is well accepted that chronic hyperglycemia is an important risk factor for myocardial infarction, and importantly, both acute and chronic hyperglycemia trigger several biochemical and electrophysiological changes resulting in an impaired cardiac contractile function [2]. Hypoglycemia first initiates repeated acute changes in cellular metabolism and then followed by cumulative long-term changes in macromolecules. The long-term changes include mainly a big amount of increases in the production of reactive oxygen species, ROS, which then induce a diabetic tissue/cell damage in several target organs including the heart [3–5]. Although oxidants are produced also in healthy tissues, increased oxidative stress plays an important role in the development of a number of diseases such as cardiovascular system disorders [6].

Accumulated evidence indicates that oxidative stress has closely been associated with diabetes and its complication including diabetic cardiomyopathy. Increased oxidative stress results also from a reduction of antioxidants/antioxidant defence system, thereby contributing to the initiation and progression of cardiac dysfunction [7]. Hyperglycemia-induced oxidative stress can also result in formation of misfolded or damaged proteins as well as changes in cellular redox status, which is closely modulated by reductants or antioxidant molecules as well as enzymes [8, 9]. Therefore, an imperfection in the cellular defence systems leads to development over time of oxidatively damaged cellular macromolecules and consequently, in part, dyshomeostasis in intracellular free Ca^2+^ [10]. In addition, it has been also shown that intracellular free Zn^2+^ level can increase rapidly in cardiomyocytes due to the mobilization of Zn^2+^ from intracellular stores mostly by ROS [11, 12].

Zinc, being an essential trace element, is vital in maintaining normal physiology and cellular functions in many cell types. Intracellularly, it is mostly bound to metalloproteins and plays a key role as an activating cofactor for many enzymes. On the cellular level, it has been demonstrated that the intracellular Zn^2+^ homeostasis is involved in signal transduction, in which Zn^2+^ acts as an intracellular mediator, similar to Ca^2+^ [13, 14]. Total Zn^2+^ in eukaryotic cells (up to 200 *μ*M) is not too different from the Ca^2+^ one with 30% localized in the nucleus, 50% in the cytosol and organelles, and the remainder associated with the proteins [15]. In cardiomyocytes, the intracellular free Zn^2+^ concentration ([Zn^2+^]_*i*_) is measured to be less than one nanomolar under physiological conditions [11, 16], similar to the one reported in HT-29 cells [17], that is, some 100-fold less than that of intracellular free Ca^2+^. Moreover, oxidants caused about 30-fold increase in intracellular free Zn^2+^ but only 2-fold in intracellular free Ca^2+^ in freshly isolated cardiomyocytes [11].

Zinc, a redox-inactive metal, has been long viewed as a component of the antioxidant network, and growing evidence points to its involvement in redox-regulated signaling via a direct or indirect regulation [18, 19]. Although there are a number of findings on cell/tissue dysfunction and its association with cellular oxidative stress level, redox signaling, and intracellular free Zn^2+^ level, very little is known about the contribution as well as the intracellular control of free Zn^2+^ in cardiomyocytes under physiological and pathophysiological conditions. Ca^2+^ release from intracellular stores mainly from sarcoplasmic reticulum (SR) via ryanodine receptors (RyR2) plays an important role in the regulation of cardiac function. Changes in the channel regulation are demonstrated to cause diastolic Ca^2+^ leakage from SR, which underlies many cardiac dysfunctions including diabetic cardiomyopathy [20]. Since redox modifications of RyR2 with direct and/or indirect action of oxidants contribute to SR Ca^2+^ leak in cardiomyocytes under many diseased-heart models [21–24], RyR2s are modulated with sulfhydryl oxidation in cardiomyocytes biphasically [25], high glucose attenuates protein S-nitrosylation via superoxide production [26], and nitric oxide (NO) mediates intracytoplasmic and intranuclear Zn^2+^ release [27], we aimed to test a hypothesis that the intracellular free Zn^2+^ changes, via increased oxidative stress and/or defective antioxidant defence system, play an important role in the development of diabetes-related alterations in RyR2 function. Under published data, we hypothesized that alterations in phosphorylation status of RyR2 are not the only type of biochemical modification in diabetic heart [20]. Shortly, diabetes is accompanied by increased oxidative stress and defective antioxidant defence system [28, 29] via increased ROS/RNS production, which can cause changes in RyR2 function as well as release of Zn^2+^ from intracellular stores [11, 12]. Accordingly, the goal of the present study was to test whether intracellular Zn^2+^ release besides Ca^2+^ release due to an increased oxidative stress/defective antioxidant defence system in cardiomyocytes under hyperglycemia mediates in part RyR2 leak and consequently diastolic dysfunction in heart from streptozotocin (STZ)-diabetic rats by using *N*-acetyl cysteine (NAC), under *in vivo* and *in vitro *approaches.

## 2. Materials and Methods

### 2.1. Experimental Rats and Diabetes Model

Male Wistar rats were used (200–250 g). Diabetes was induced in diabetic group as previously described [20]. One week after injection of STZ, blood glucose level was measured and rats with at least 3-fold higher level of blood glucose than preinjection level were used in the experiments as diabetic animals (DM group). Diabetic animals, 4 weeks after diabetes confirmation, received either *N*-acetyl cysteine (NAC; 150 mg/kg, daily and intragastrically, DM + NAC group) or vehicle (saline) for 4 weeks in an identical fashion, while nondiabetic rats (CON group) received saline alone. All rats had free access to standard chow and water. All animals were handled in accordance to the Guide for the Care and Use of Laboratory Animals published by the US National Institutes of Health (NIH publication number 85-23, revised 1996). The protocol was approved by the Ankara University Experimental Animals Ethics Committee, and approval reference number is 2011-115-449.

### 2.2. Assessment of Oxidative Stress/Antioxidant Status in Plasma and Heart Homogenates

Lipid peroxides, derived from polyunsaturated fatty acids, are unstable and decompose to form a complex series of compounds, which include reactive carbonyl compounds, such as MDA. Plasma lipid peroxidation was determined by measuring malondialdehyde (MDA) level with thiobarbituric acid reactive substances (TBARS) assay kit (Cayman Chemical Company). The MDA-TBA adduct was formed under high temperature and acidic condition was measured colorimetrically at 530–540 nm.

Plasma glutathione status was determined by measuring reduced glutathione (GSH) and oxidized glutathione (GSSG) levels, carried out with glutathione assay kit (Cayman Chemical Company) containing 2-(N-morpholino)ethanesulfonic acid buffer, GSSG standard, enzyme mixture, and 5,5′-dithiobis(2-nitrobenzoate). Levels of GSH and GSSG were calculated using reduced glutathione standard and the results were expressed as *μ*mol/L. Detection limits for GSH and GSSG were 0.1 *μ*mol/L and 0.001 *μ*mol/L, respectively, while intraassay coefficients of variation for GSH and GSSG were 0.96% and 6.45%, respectively.

To prepare heart homogenates, first frozen hearts were pulverized at liquid N_2_ temperature and then homogenized [30]. Protein contents in homogenates were analyzed by using the Bradford method (Bio-Rad). Bovine serum albumin was used as a protein standard. Protein oxidation level in heart homogenates was determined as described previously [31].

Total sulfhydryl (SH) and acid-soluble sulfhydryl (total thiol and free thiol) groups of proteins were estimated with Ellman's reagent as described previously [31]. Shortly, heart homogenates were thawed and lysed in 0.2 M Tris/HCl buffer, pH 8.1, containing 2% sodium dodecylsulfate. For determination of total SH groups, 0.05 mL of aliquots of cell lysates was mixed with 0.8 mL of distilled water and 0.1 mL of 2 mM 5,5′-dithiobis-(2-nitrobenzoic acid). Absorbances of the supernatants were read at 412 nm (Shimadzu UV-120-02 spectrophotometer). After correction of the absorbances with sample and reagent blanks, levels of SH groups in each sample were calculated employing an extinction coefficient of 1.31 mM^−1^·mm^−1^. To determine level of acid-soluble SH groups, 0.7 mL aliquots of heart homogenate lysates were mixed with 0.35 mL of 20% trichloroacetic acid (TCA) and final precipitates were washed with 0.2 mL of 20% TCA in a similar manner and the supernatants were combined and brought to pH 8 with NaOH. The total SH levels of the supernatants were measured as described above for free SH measurement.

### 2.3. Electron Microscopy

Histological examination was performed as described elsewhere. For electron microscopy evaluation, samples were fixed in 2.5% glutaraldehyde in phosphate buffer for 2–4 h at 4°C and postfixed in 1% osmium tetroxide. Following samples dehydration through graded alcohol concentrations (50%, 75%, 96%, and 100%), the heart tissues were embedded into Araldite 6005. The embedded samples were sliced at a thickness of 4–6 *μ*m using a Leitz-1512 microtome. Sections were stained with uranyl acetate-lead citrate for examination by using a Leo 906 E transmission electron microscopy.

### 2.4. Isolated Langendorff-Perfused Hearts

Hearts were isolated and perfused as described previously [30]. Briefly, isolated hearts were electrically stimulated (DCS, Harvard Instruments) at 300 beats/min by a square wave of twice the threshold voltage of 1.5 ms duration. Hearts were perfused for a total of 50–60 min and functional parameters were determined at 40 min. The left ventricular end-diastolic pressure (LVEDP) changes of isolated hearts were measured.

### 2.5. Isolation of Ventricular Cardiomyocytes

Cell isolation was performed as described elsewhere [31]. Briefly, rats were anaesthetized using sodium pentobarbital (30 mg/kg, intraperitoneal) and ventricles were removed from rapidly excised hearts and minced into small pieces and gently passed through a nylon mesh. Following collagenase digestion, dissociated cardiomyocytes were washed with collagenase-free solution. Subsequently Ca^2+^ in the medium was gradually increased to a final concentration of 1.3 mM. Cells were kept in this solution at 37°C and only Ca^2+^ tolerant cells were used in the experiments.

### 2.6. Measurement of Cytosolic Zn^2+^ and Ca^2+^ in Resting and Electrically Stimulated Cells

Fluorescence changes were recorded using microspectrophotometer and FELIX software (PTI, Lawrenceville, NJ, USA) as described previously [12]. Cardiomyocytes were loaded with cell-permeable acetoxymethoxy derivatives of FluoZin-3 or Fluo-3 (FluoZin-3-AM or Fluo-3 AM; 35–40 min incubation) and excited at 0.2 Hz by field stimulation using two platinum electrodes positioned on either side of the recording chamber. The basal levels of [Zn^2+^]_*i*_ and [Ca^2+^]_*i*_ were measured from Fura-2 loaded (4 *μ*M Fura-2 AM) cardiomyocytes at room temperature as described previously [11]. The transient fluorescence changes under electrical stimulation, which are estimated from the difference between peak and basal levels, were detected. All experiments were performed at room temperature. Cells were superfused continuously with HEPES-buffered solution as used for confocal imaging. Cells were excited at 488 nm for FluoZin-3 and at 480 nm for Fluo-3, and emissions were recorded at 520 and 515 nm, respectively. Cells showing a significant fluorescence decrease during the 5 min equilibration period were eliminated. The amplitude of Zn^2+^ or Ca^2+^ transients was stable within 10% during experimental period.

### 2.7. Confocal Measurement of Zn^2+^- and Ca^2+^-Sparks

Short-lived, tiny, and localized light emissions (named sparks) were recorded from different cardiomyocytes after 35–40 min incubation with FluoZin-3-AM or Fluo-3 AM, respectively, as described previously [12]. Loaded cells were transferred to the experimental chamber mounted on the stage of a Leica TCS SP5 laser scanning microscope. Experiments were conducted on quiescent cardiomyocytes. FluoZin-3 or Fluo-3 was excited at either 485 or 506 nm, and emission was collected at either 535 or 526 nm, respectively. In some experiments, recordings were obtained with HEPES-buffered solution supplemented with either zinc ionophore pyrithione (ZnPT; 1 or 10 *μ*M), with the subsequent addition of 50 mM N,N,N′,N′-tetrakis(2-pyridylmethyl) ethylenediamine (TPEN) to bring the intracellular free Zn^2+^ level to zero. Experiments were performed at room temperature.

Sparks were initially detected with ImageJ (SparkMaster, plug-in) which is an open access programme (http://rsb.info.nih.gov/ij) and then were manually selected. The parameters of fluorescence changes such as peak amplitude (Δ*F*/*F*_0_, where Δ*F* = *F* − *F*_0_; *F* was identified as local maximum elevation of fluorescence intensity over basal level, *F*_0_), time to peak (TP), frequency, full duration at half-maximum, or rise time (FDHM) were calculated automatically by using ImageJ programme.

### 2.8. Western Blot Analysis

For preparation of tissue homogenates, frozen heart samples from left ventricle were crushed at liquid N_2_ temperature and then homogenized to measure the phosphorylation and protein levels of contractile machinery complex as described previously [20] (CaMKII, phospho-CaMKII-Thr286, PKA, phospho-PKA-Thr198, FKBP12.6, RyR2, phospho-RyR2-Ser^2808^, phospho-NF*κ*B, NF*κ*B, and *β*-actin were identified using specific antibodies with recommended dilutions of either Santa Cruz biotechnology (USA) or Badrilla Ltd. (UK) companies). Density analysis of protein bands was performed using ImageJ programme.

Similar Western blot analysis was also performed in the diabetic rat cardiomyocytes from left ventricle incubated with *N*-acetyl cysteine (NAC; 1 mM) for 1 h at 37°C.

### 2.9. Chemicals and Data Analysis

Unless otherwise stated, all chemicals used were purchased from Sigma (Sigma-Aldrich Chemie, Steinheim, Germany).

Groups were tested and compared using one-way ANOVA and Tukey post hoc test. Values of *P* < 0.05 were taken as statistically significant. Significance levels are given in the text, and data are presented as means ± SEM.

## 3. Results

### 3.1. General Characteristics of the Experimental Animals

**Figure 1:**
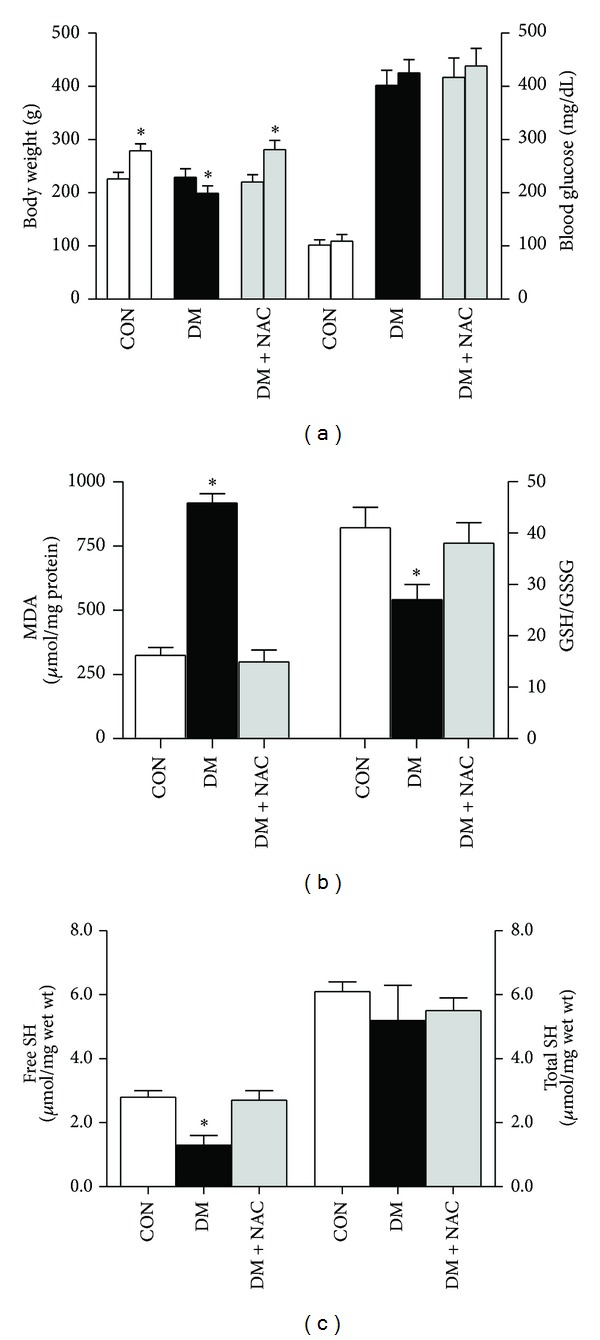
Body weight, blood glucose, and oxidative stress markers of normal rats (CON group) and diabetic (DM) rats treated with *N*-acetyl cysteine (DM + NAC group) or untreated DM rats. (a) Body weight (left) and blood glucose levels (right) in the rats both at the beginning (first column) and 5th week end (second column) of experimental period. (b) The levels of lipid peroxidation marker malondialdehyde, MDA, (left) and the reduced glutathione to oxidized glutathione (GSH/GSSG) ratio (right), and (c) total (right) and free (left) thiol (SH) levels measured in heart homogenates. Bar graphs represent mean ± SEM values and number of rats in groups; *n*_CON_ = 8, *n*_DM_ = 7, and *n*_DM+NAC_ = 6, respectively. Significant at *P < 0.05, due to comparison between 1st and 5th weeks.

A single administration of streptozotocin, STZ, to rats induced diabetic symptoms compared to the aged-matched controls including body weight loss (199 ± 14 g versus 279 ± 13 g; number of rats 32 versus 24) and marked increase in blood glucose level (425 ± 25 mg/dL versus 109 ± 13 mg/dL). The rats from an antioxidant *N*-acetyl cysteine (NAC)-treated diabetic group (DM + NAC group) gained body weight similar to the controls (281 ± 17 g versus 279 ± 13 g; *n* = 11 rats) although they had high blood glucose level at the end of the experimental period (438 ± 33 mg/dL versus 109 ± 13 mg/dL) (Figure 1). In order to demonstrate whether there is hypertrophy in the diabetic rat hearts and to avoid the possible insensitive heart to body weight ratio measurement, we measured the cell capacitance in the isolated cardiomyocytes and compared the values between the groups. In STZ-induced diabetic rat heart during 7-8 weeks, hypertrophy has not been measured (data not shown).

### 3.2. Ultrastructure, Oxidative Stress, and Redox Status in Diabetic Rat Heart

The extent of hyperglycemia-induced oxidative stress, lipid peroxidation level evaluated by MDA analysis in the heart homogenate, was significantly decreased in *N*-acetyl cysteine (NAC)-treated diabetic group (298 ± 47 *μ*mol/mg protein) when compared to untreated diabetic group (917 ± 36 *μ*mol/mg protein), which was also significantly higher compared to that of the control group (324 ± 31 *μ*mol/mg protein), suggesting both increased oxidative stress and an antioxidant role of NAC during hyperglycemic stress in heart. The extent of altered redox status in the heart was detected by measuring a ratio of GSH to GSSG (GSH/GSSG). This ratio was found to be significantly lower in the diabetic group (27 ± 3) when compared to both the controls (41 ± 4) and NAC-treated diabetics (48 ± 9), suggesting the altered cellular redox status in the heart during hyperglycemic stress. Furthermore, we found that the oxidation level of protein-SH (thiol) was significantly higher in diabetic rat heart homogenate (free thiol level: 1.3 ± 0.3 *μ*mol/mg wet wt) compared to that of the control (free thiol level: 2.8 ± 0.2 *μ*mol/mg wet wt) (Figure 1). Free protein-thiol level in the homogenate from NAC-treated diabetic group (2.7 ± 0.3 *μ*mol/mg wet wt) was found to be significantly lower compared to that of untreated diabetic group. Furthermore, total protein-thiol level was not affected by either diabetes or NAC treatment (data not shown). We, therefore, demonstrated that there is marked increase in oxidative stress in the heart of diabetics besides a high circulatory level of lipid peroxidation [32] from STZ-induced diabetic rat, and it can be controlled to normal level by NAC treatment, which is confirmed also with previously published data [33–35].

**Figure 2:**
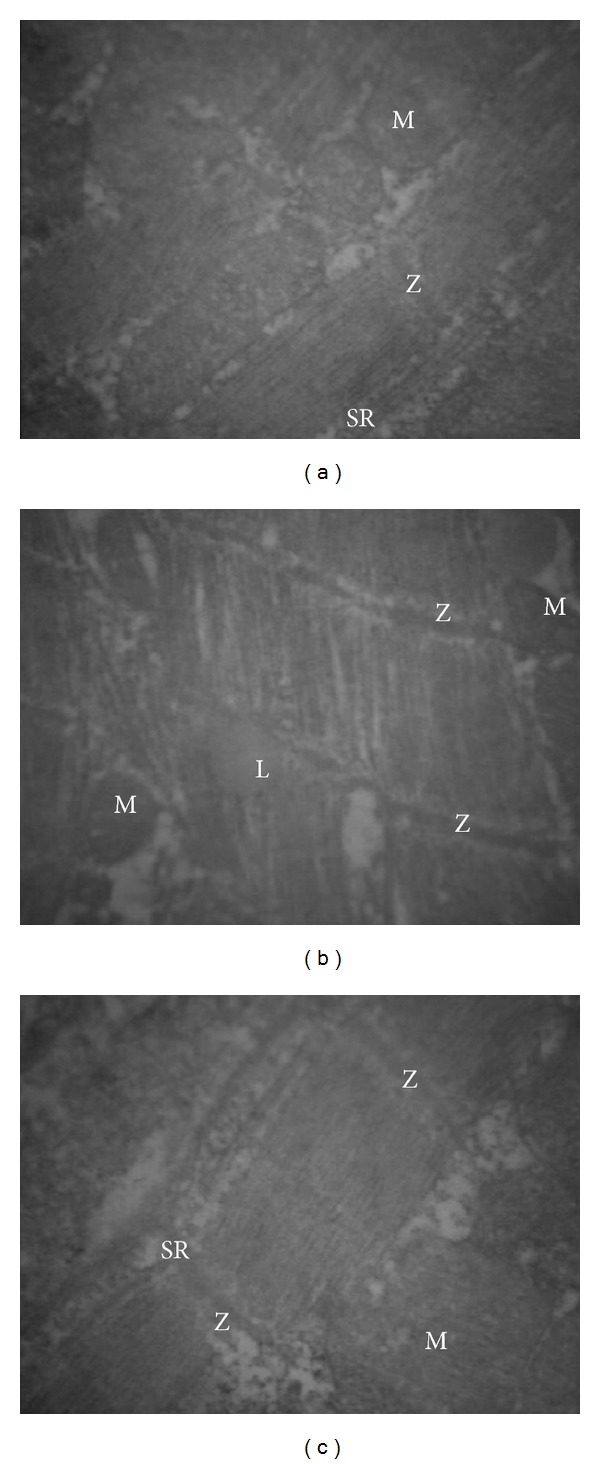
Morphological findings in experimental rat hearts. Micrographs of left ventricular cross section through control (CON), diabetic (DM), and *N*-acetyl cysteine (NAC)-treated diabetic (DM + NAC group; 150 mg/kg, daily and intragastrically, for 4 weeks) groups, respectively, obtained by electron microscopy. (a) There is no sign of any tissue necrosis in CON group. Marked alterations in myofilaments and sarcoplasmic reticulum (SR), destruction in Z-lines (Z), loss of cristae and granular matrix in mitochondria (M), and also increased numbers of lipid droplets (L) are seen in DM group (b) while these alterations have almost disappeared with NAC treatment (c); magnifications are ×12,390, ×10,000, and ×12,990 for (a), (b), and (c), respectively.

Although diabetic rats did not show abnormal behaviour or any sign of heart failure as well as any signs of any tissue necrosis, as shown previously [36], the ultrastructure of diabetic rat heart showed various morphological changes including a loss in cardiomyocyte diameter, alterations in myofilaments and Z-lines of myofibers, myofibrillar degeneration, and destruction and loss of myofibrils over sarcomere lengths when compared to control hearts (Figures 2(a) and 2(b)). In the diabetic group most of the mitochondria of the cardiomyocytes showed loss of cristae and granular matrix and also increased numbers of lipid droplets (Figure 2(b)). NAC treatment normalized fully these alterations in the myofilaments (Figure 2(c)).

For quantification of electron microscopy findings among diabetic and nondiabetic rat hearts, we investigated only the lipid droplets and observed basically that the number and size of the lipid droplets in the diabetic group were significantly higher than that of the control group (~40% and ~15% for diabetic versus control group) while these changes have fully disappeared in the NAC-treated group. The lipid quantification of cardiac tissue was performed by using oil red O staining which revealed severe lipid accumulation in the heart. The lipid contents were expressed as lipid area normalized to total investigated tissue area.

### 3.3. Cardiac Function of Diabetic Rat Heart

**Figure 3:**
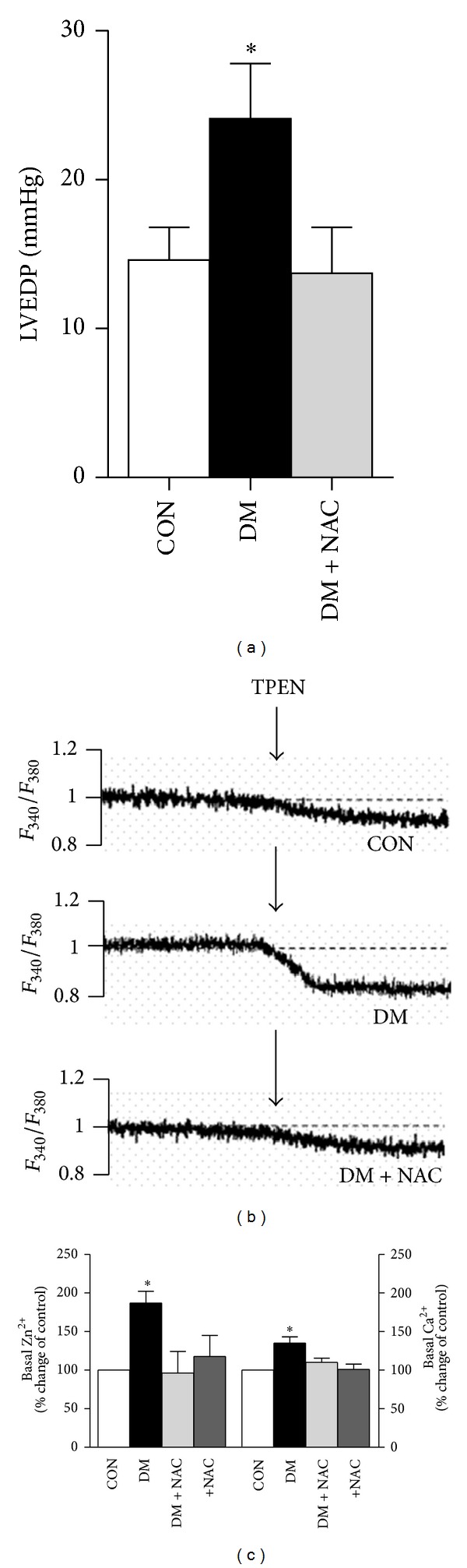
*N-*acetyl cysteine (NAC) treatment normalizes diastolic function of the heart due to normalization of increased levels of basal Ca^2+^ and Zn^2+^. (a) Left ventricular end diastolic pressure, LVEDP, changes among the groups are given for controls (CON), diabetics (DM), and NAC-treated diabetics (DM + NAC group; 150 mg/kg, daily and intragastrically, for 4 weeks). (b) Representative traces showing how basal levels of intracellular free Ca^2+^ and Zn^2+^can be demonstrated by using fluorescence changes following TPEN application in cardiomyocytes loaded with Furo-AM. The average values of basal intracellular free Zn^2+^ (left) and Ca^2+^ (right) are given in (c). Bar graphs represent mean ± SEM values. In here, a fourth group is NAC-incubated diabetic cells (+NAC; 1 mM, for 1 h at 37°C). The numbers of animals/cells for each group as well as for each protocol, at least used, are the following; *n*_CON_ = 5, *n*_cell_ = 15; *n*_DM_ = 5, *n*_cell_ = 20; *n*_DM+NAC_ = 4, *n*_cell_ = 12; *n*_+NAC_ = 5, *n*_cell_ = 19 for CON, DM, DM + NAC, and +NAC groups, respectively. Significant at *P < 0.05 versus CON group.

We, previously, showed that STZ injection in rats either short- (4-5 weeks) or long-period (8–12 weeks) experimental durations induced marked several alterations in electrical and mechanical parameters of heart [12, 29, 37, 38]. Left ventricular systolic and diastolic dysfunctions are among these alterations. In the present study, since we aimed to investigate a possible contribution of intracellular free Zn^2+^ release besides that of Ca^2+^ release due to an increased oxidative stress under hyperglycemia, we first examined diastolic function of heart from diabetic rat and compared it to those of control and *N*-acetyl cysteine (NAC)-treated rats. Rats following 5-6 weeks of STZ injection developed increased diastolic dysfunction (Figure 3(a)), as evidenced by about 80% increase in left ventircular end diastolic pressure (LVEDP). NAC treatment of diabetic rats for 4 weeks induced a full normalization in LVEDP.

### 3.4. Basal Levels of Intracellular Free Zn^2+^ and Ca^2+^ in Cardimyocytes from Diabetic Rats

To identify and compare basal intracellular free Zn^2+^ level in diabetic rat cardiomyocytes with that of the control, we used a ratiometric fluorescence dye, Fura-2 AM. In order to compare the individual data, we normalized the initial fluorescence values to obtain a common ratio in all cardiomyocytes, and then we used TPEN responses for minimum and maximum fluorescence changes (Δ*F*_340/380_). The original fluorescence changes for basal [Zn^2+^]_*i*_ level are given in Figure 3(b). The data are given as percentage changes in order to compare diabetic and *N*-acetyl cysteine (NAC)-treated diabetic groups with the control group. As can be seen in Figure 3(b), the addition of the membrane-permeant Zn^2+^ chelator TPEN (50 *μ*M) caused a rapid decrease in fluorescence ratio (Δ*F*_340/380_) to a level lower than that of initial value, verifying that the initial observed fluorescence ratio is attributable to intracellular basal free Zn^2+^ level. This decrease is bigger in the diabetic group compared to that of the control group while it is almost the same level in the NAC-treated diabetic group and the control group as well. As can be seen in the same bar graphs, the fluorescence change with respect to the basal level of intracellular free Ca^2+^ is significantly higher in the diabetic cardiomyocytes compared to those of both the control and NAC-treated diabetic groups. The present data demonstrate that both increased intracellular basal free Zn^2+^ and Ca^2+^ levels are dependent on increased oxidative stress and decreased antioxidant-defence system in the hyperglycemic rats.

To test directly whether increased oxidative stress can induce simultaneous increases in basal levels of both intracellular free Zn^2+^ and Ca^2+^ of diabetic rat cardiomyocytes, we incubated cardiomyocytes from diabetic rats with NAC (1 mM, for 1 h at 37°C) before loading the cells with Fura-2 AM. As can be seen from Figure 3(c), NAC incubation of diabetic cardiomyocytes (+NAC group) induced a similar change in the fluorescence ratios related to basal levels of both intracellular free Zn^2+^ and Ca^2+^. These groups of experiments confirm also that diabetes can induce significant increases not only in basal Ca^2+^ level but also in basal Zn^2+^ level, at most, due to increased reactive oxygen species, ROS.

### 3.5. Local Intracellular Releases of Both Zn^2+^ and Ca^2+^ in Diabetic Rat Cardiomyocytes

**Figure 4:**
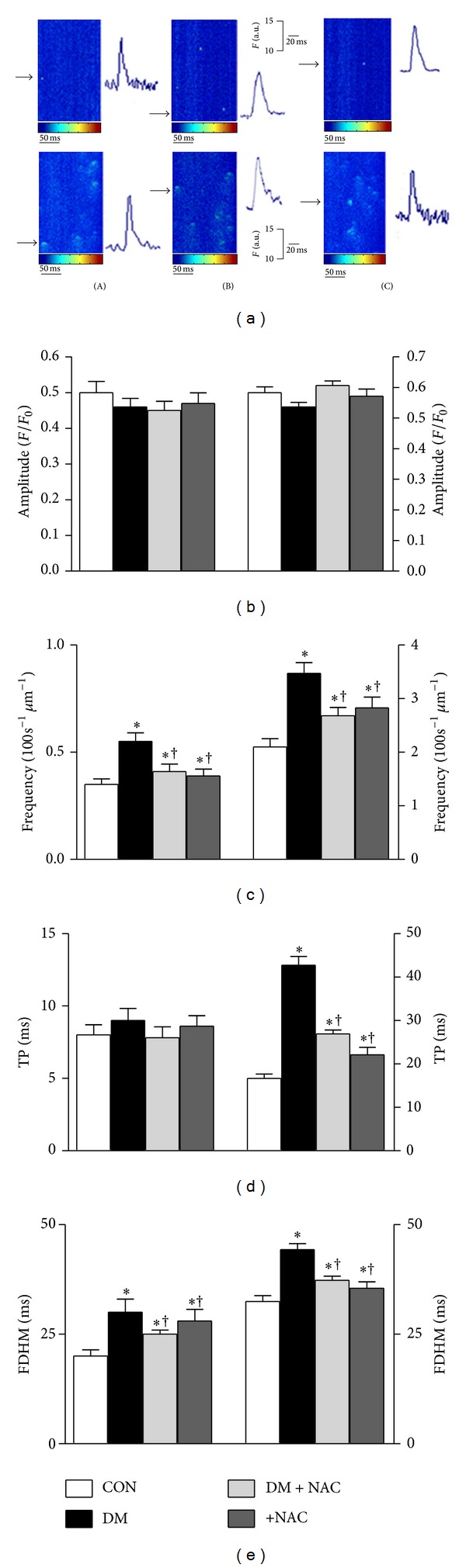
*N*-acetyl cysteine (NAC) treatment/incubation of diabetic rats/diabetic cells ameliorates altered parameters of both Ca^2+^ and Zn^2+^ sparks. (a) Representative line-scan images of freshly isolated cardiomyocytes (CON: control group, DM: diabetic group, DM + NAC: *N*-acetyl cysteine-treated DM group (150 mg/kg, daily and intragastrically, for 4 weeks), and +NAC: NAC-incubated diabetic cells; 1 mM, for 1 h at 37°C). The peak intensity and frequency of either FluoZin-3 (left) or Fluo-3 (right) recorded in the groups are given in (b) and (c), respectively. Time to peak amplitude (TP) and full duration at half-maximum (FDHM) of both fluorescence changes are given in (d) and (e), respectively. Bar graphs represent mean ± SEM values (*n*_rat_ = 5, *n*_cell_ = 44, *n*_spark_ = 150; *n*_rat_ = 5, *n*_cell_ = 55, *n*_spark_ = 165; *n*_rat_ = 5, *n*_cell_ = 38, *n*_spark_ = 135 in CON, DM, DM + NAC, and +NAC, resp.). Significant at *P < 0.05 versus CON; ^†^ *P* < 0.05 versus DM.

Recently, we have demonstrated that there are local tiny Zn^2+^ releases (named as Zn^2+^ sparks) in resting quiescent cardiomyocytes isolated from 3-month-old male rats and loaded with a Zn^2+^-specific fluorescence dye, FluZin-3, which can be visualized in a similar manner to known Ca^2+^ sparks [12]. The contributions of elementary Zn^2+^ release similar to Ca^2+^ release, either Zn^2+^ or Ca^2+^ sparks, to increased basal levels of both Zn^2+^ and Ca^2+^ (mentioned in previous section) and altered Zn^2+^/Ca^2+^ contents of SR (of which, in part, present a function of SR Ca^2+^ release channels, RyR2) were examined and monitored the differences in parameters of these sparks among the diabetic and control groups. Representative line-scan images of of both type of sparks from control (CON group), diabetic (DM group), and *N*-acetyl cysteine (NAC)-treated diabetic (DM + NAC group) rats (individual Zn^2+^ or Ca^2+^ sparks images as *x* versus *t*) are displayed in Figure 4(a). The maximum fluorescence intensity, determined as Δ*F*/*F*_0_ at the peak of either Zn^2+^ or Ca^2+^ sparks (Figure 4(b); left and right, resp.), and spontaneous Zn^2+^/Ca^2+^-spark frequencies (Figure 4(c); left and right, resp.) were calculated from individual gamma distribution function fits. The maximum FluoZin-3 intensity as well as Fluo-3 intensity was not significantly different among the DM and CON groups. On the other hand, we observed marked increases in their occurrence frequency of both fluorescences during diabetes. Furthermore, time to peak (TP) of maximum FluoZin-3 intensity was found to be similar among the DM and CON groups; TP value for maximum Fluo-3 was markedly prolonged in DM group compared to that of the CON group (Figure 4(d); left and right, resp.). Moreover, the full duration at half-maximum (FDHM) of both FluoZin-3 and Fluo-3 was also found to be significantly prolonged in DM group with respect to age-matched CON group (Figure 4(e); left and right, resp.). *N*-acetyl cysteine (NAC)-treatment of the diabetic rats (DM + NAC group) for 4 weeks significantly prevented diabetes-induced all changes in both Zn^2+^ and Ca^2+^ sparks parameters.

Of note, as shown in Figures 4(b)–4(e), essentially similar results on the parameters of both Zn^2+^ and Ca^2+^-sparks were obtained in cardiomyocytes isolated from diabetic rats after 1 h incubation with 1 mM NAC at 37°C (+NAC group).

### 3.6. Regulation of Both Zn^2+^ and Ca^2+^ Transients with an Antioxidant, *N*-Acetyl Cysteine

**Figure 5:**
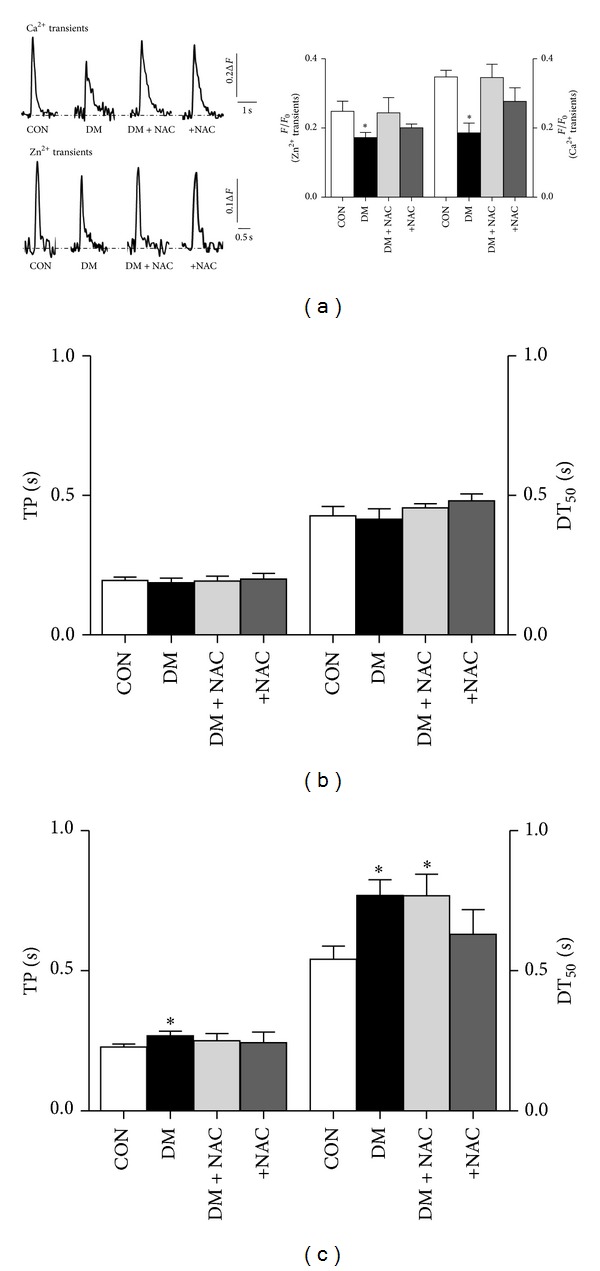
Altered intracellular global either Ca^2+^ or Zn^2+^ changes in cardiomyocytes are normalized with either *N*-acetyl cysteine (NAC)-treatment of diabetic rats or NAC-incubation in diabetic cardiomyocytes. (a) *Inset:* representative Ca^2+^ or Zn^2+^ transients in freshly isolated cardiomyocytes loaded with either FluoZin-3 (low) or Fluo-3 (up) and field-stimulated at 0.2 Hz (left). The peak amplitude of the fluorescences related to either global Ca^2+^ or Zn^2+^ (the transient changes) is given as *F*/*F*_0_. The effect of NAC treatment of diabetic rats (DM + NAC group: 150 mg/kg, daily and intragastrically, for 4 weeks) or NAC incubation of diabetic cardiomyocytes (+NAC group; 1 mM, for 1 h at 37°C) on the time to peak fluorescence, TP (left), and half-decay time, DT_50_ (right), of fluorescences either FluoZin-3 (b) or Fluo-3 (c). Bars represent mean ± SEM for controls (CON; *n*_rat_ = 5, *n*_cell_ = 21) and diabetics (DM; *n*_rat_ = 5, *n*_cell_ = 28) as well as DM + NAC (*n*_rat_ = 6, *n*_cell_ = 34) and +NAC (*n*_rat_ = 4, *n*_cell_ = 22) groups, respectively. Significant at *P < 0.05 versus CON.

To further understand the effects of both diabetes and NAC treatment of diabetes on the distributions of both intracellular free Zn^2+^ and Ca^2+^ changes in isolated cardiomyocytes, we performed some additional experiments to monitor intracellular transient changes of either intracellular free Zn^2+^ or Ca^2+^ elicited by electrical-field stimulation.  Figure 5(a) (left) shows original recordings of Zn^2+^ and Ca^2+^ transients elicited in control, diabetic, and *N*-acetyl cysteine (NAC)-treated (4 weeks) diabetic rat cardiomyocytes as well as NAC-incubated diabetic cardiomyocytes for 1 h with 1 mM NAC at 37°C. The averaged peak fluorescence amplitudes of both FluoZin-3 and Fluo-3 as *F*/*F*_0_ were significantly smaller in diabetic cells than in control cells (Figure 5(a), right) while their amplitudes in either NAC-treated diabetic rat cardiomyocytes or NAC-incubated diabetic cardiomyocytes were found to be similar to those of the controls. The time to peak amplitude (TP) and the half-time for recovery (DT_50_) of transient changes of FluoZin-3 were found to be similar between diabetic and control groups as well as NAC-treated or NAC-incubated diabetics (Figure 5(b)). In addition, the similar parameters of transient changes of Fluo-3 were significantly prolonged in diabetic groups compared to that of the controls. These changes were also significantly prevented with either NAC treatment of diabetic rats or NAC incubation of diabetic cardiomyocytes for 1 h with 1 mM at 37°C (Figure 5(c)).

### 3.7. Biochemical Analysis of Cardiac Ryanodine Receptors, RyR2 and Calstabin2

**Figure 6:**
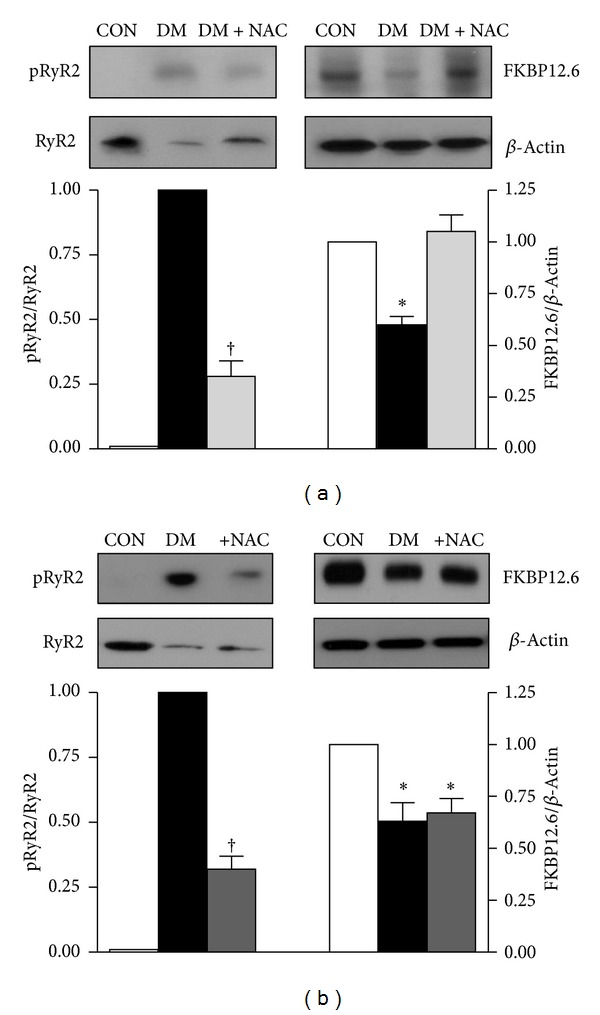
Effect of *N-*acetyl cysteine (NAC) treatment of diabetic rats or NAC incubation of diabetic cardiomyocytes on hyperphosphorylation and protein levels of SR Ca^2+^ release channel (RyR2) and its accessory proteins. (a)* Top*: representative Western blot images for phospho-RyR2-Ser^2808^ (pRyR2; upper left band), and FK-binding protein, calstabin2 (FKBP; upper right band), RyR2 (lower left band), and *β*-actin (lower right band). *Bottom*: quantification for the ratio of pRyR2 to RyR2 and FKBP to *β*-actin. Data presented in (a) are obtained from control (CON), diabetic (DM), and NAC-treated diabetic (DM + NAC; 150 mg/kg, daily and intragastrically, for 4 weeks) rat heart homogenates. (b) *Top*: representative Western blotting for phospho-RyR2-Ser^2808^ (pRyR2; upper left band), calstabin2, (FKBP; upper right band), RyR2 (lower left band), and *β*-actin (lower right band). *Bottom*: quantification for the ratio of pRyR2 to RyR2 and FKBP to *β*-actin. Data presented in (b) are obtained from controls (CON), diabetics (DM), and NAC-incubated diabetic cardiomyocytes (+NAC group: 1 mM, for 1 h at 37°C). Bar graphs represent mean ± SEM (*n*_rats_ = 6–8 for each protocol/group). Significant at *P < 0.05 versus CON and ^†^ *P* < 0.05 versus DM.

It has been previously shown that the mechanisms underlying the dysfunction of RyR2 in diabetes include, in part, a hyperphosphorylation of RyR2 due to both high phosphorylation levels of both protein kinase A (PKA) and Ca^2+^-calmodulin kinase II (CaMKII) under hyperglycemia [12]. The phosphorylation level of RyR2 in diabetic and control rat left ventricular heart tissue was evaluated by using specific antibodies directed against RyR2 and phosphorylated RyR2 (Figure 6(a), left). Total RyR2 in the diabetic group was about 64% less than that of the control group as estimated from the Western blot bands. From the band-intensity analysis, there was a strong evidence for the RyR2 phosphorylation in diabetic rat heart homogenates while no detectable band in the heart homogenates of the control group. The amount of calstabin2 (FKBP12.6) was decreased by 40% in the diabetic rat heart compared to that of the control (Figure 6(a), right). There was no significant difference in the protein levels of actin in diabetic and control groups (Figure 6(a), right). In addition, these parameters were found to be markedly prevented by NAC treatment of diabetic rats for 4 weeks.

For an assessment of a direct action of antioxidant NAC, we first incubated freshly isolated cardiomyocytes from diabetic rats with 1 mM NAC for 1 h at 37°C and then performed the similar Western blot analysis for pRyR2, RyR2, FKBP12.6. Incubation of diabetic cardiomyocytes with 1 mM NAC significantly declined hyperphosphorylation level in RyR2, while there was no effect of its depressed protein level (Figure 6(b), left). Similarly, NAC incubation did not affect the decreased FKBP12.6 protein level in diabetic cardiomyocytes (Figure 6(b), right).

### 3.8. Direct Effects of External Zn^2+^ on RyR2 Macromolecular-Complex of Ventricular Heart Tissue

**Figure 7:**
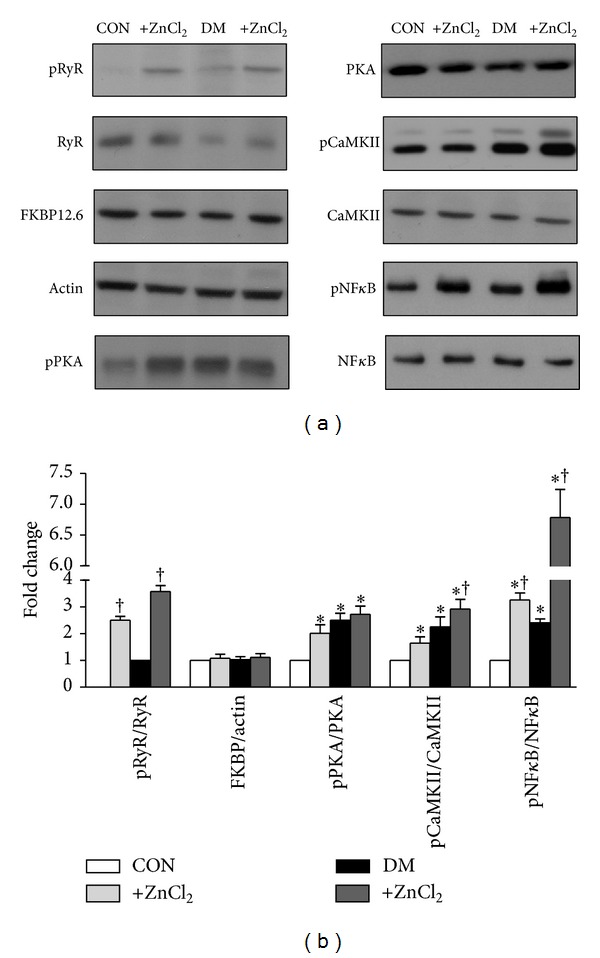
Direct effects of external Zn^2+^ on RyR2 macromolecular complex of ventricular heart tissue. (a)* Top*: representative Western blot images for phospho-RyR2-Ser^2808^ (pRyR2), FK-binding protein, calstabin2 (FKBP), protein kinase A (PKA), phosphorylated PKA (phospho-PKA-Thr198), Ca^2+^-calmodulin-dependent kinase (CaMKII), phosphorylated CaMKII (phospho-CaMKII-Thr286), nuclear factor kappaB (NF-*κ*B), phosphorylated NF-*κ*B (phosoho-NF-*κ*B), *β*-actin. (b) Quantification for the ratio of pRyR2 to RyR2, FKBP to *β*-actin, pPKA to PKA, pCaMKII to CaMKII and pNF-*κ*B to NF-*κ*B. Data presented in (b) obtained from control (CON), 1 *μ*M ZnCl_2_ incubated control (+ZnCl_2,_ light gray), diabetic (DM) and 1 *μ*M ZnCl_2_ incubated diabetic (+ZnCl_2_, dark gray) rat heart homogenates. Bar graphs represent mean ± SEM (*n*_rats_ = 7–9 for each protocol/group). Significant at *P < 0.05 versus CON and ^†^ *P* < 0.05 versus DM.

The intracellular free Zn^2+^ distribution is linked to redox metabolism despite Zn^2+^ itself not being redox-active and generally Zn^2+^-proteins being redox-inert [39, 40]. Therefore, since we previously have shown a mediation of rapid, large, and selective elevation of intracellular free Zn^2+^ through the modulation of the redox status of intracellular protein thiols, in here, we aimed to examine the effects of external ZnCl_2_ incubation (10 *μ*M) of heart homogenates from both control and diabetic groups rats for 20–30 min at 37°C on members of RyR2-macromolecular complex. As can be seen from Figures 7(a) and 7(b), total protein levels of RyR2 and its accessory proteins, FKBP12.6, PKA, and CaMKII, were not affected with either 1 *μ*M (data not shown) or 10 *μ*M ZnCl_2_ incubation of left ventricular heart homogenates from both group rats. However, we measured significantly increased phosphorylation levels of RyR2, PKA, and CaMKII with these two ZnCl_2_ incubations in a concentration-dependent manner. The last data further support the hypothesis that Zn^2+^ disbalance results in a signaling disbalance caused by a local surplus of Zn^2+^ interfering with cellular signaling networks.

### 3.9. Relation between Intracellular Zn^2+^ and Nuclear Factor Kappa B (NF-*κ*B) Activation

There are multiple studies with different conclusions regarding whether NF-*κ*B is protective or detrimental for heart function although its important role in cardiac pathology [41, 42] has been demonstrated. This disagreement is not surprising considering the complexity of NF-*κ*B signaling that involves multiple components and regulation at several steps. Furthermore, NF-*κ*B is a pleiotropic transcription factor that receives signals from multiple pathways including different important modulators of cardiac remodeling. To asses a possible direct role of intracellular Zn^2+^ on NF-*κ*B, we tested the effect of external ZnCl_2_ incubation (10 *μ*M) of heart homogenates from both control and diabetic groups rats for 20–30 min at 37°C, similar to previous section. As can be seen from Figures 7(a) and 7(b), total protein level of NF-*κ*B was not affected with 10 *μ*M ZnCl_2_ incubation of left ventricular heart homogenates from both control and diabetic group rats. However, the activation levels of NF-*κ*B in both group rat heart homogenates were significantly increased (~3-fold in both groups) after ZnCl_2_ incubations. This last data further supports the hypothesis that although cardiac remodeling is associated with increased oxidative stress, inflammation, and activation of hormonal systems under hyperglycemia, increased intracellular ZnCl_2_-mediated NF-*κ*B activation seems to be a distinct fact among the others under high Zn^2+^ exposure of heart.

## 4. Discussion

This study on *N*-acetyl cysteine (NAC) effect in STZ-induced diabetes in rats reports a significant role of cellular antioxidant-defence enhancement on preservation of diastolic dysfunction via regulation of not only diastolic Zn^2+^ but also diastolic Ca^2+^ due to a prevention of RyR2-leak in diabetic rat heart. At first, we confirmed a defective intracellular Zn^2+^ signaling, besides previously shown Ca^2+^ signaling [20], in part, due to increased oxidative stress/depressed antioxidant defence in diabetic cardiomyocytes, with lower amplitude of Zn^2+^ transients as well as markedly increased diastolic levels of both Zn^2+^ and Ca^2+^ since all these alterations could be prevented with either NAC treatment of diabetic rats or NAC-treatment of diabetic cardiomyocytes. Our electron microscopy data further corroborated these findings fully. Furthermore, we clearly established that these defects in diabetic cardiomyocytes could be attributed to anomalous RyR2 behavior, as revealed by the spatiotemporal properties of both Zn^2+^ and Ca^2+^ sparks that especially exhibited slower kinetics and higher frequencies. The reduced amount of RyR2 and FKBP12.6 levels as well as a marked hyperphosphorylation of RyR2 could be responsible for most of these observations, in part, due to a disbalanced ratio of oxidants/antioxidant-defence. Moreover, the initial findings of this study indicate that supplementation with NAC improves the general status of diabetic rats, with an effect on body weight gain and hyperglycemia, protecting diastolic function, and helping to maintain baseline myocardial mechanics.

### 4.1. Antioxidant *N*-Acetyl Cysteine Prevents RyR2 Leak and Plays an Important Role in Diastolic Dysfunction via Increased Levels of Both Zn^2+^ and Ca^2+^ in Diabetic Heart

The present study shows that systemic antioxidant treatment of diabetic rats preserves changes in both Zn^2+^ and Ca^2+^ regulation in diabetic cardiomyocytes without any effect on high blood glucose level and restores normal macromolecular complex composition and function of the RyR2 channels in diabetic rat hearts. There is associated restoration of myocardial diastolic function and reverse structural remodeling of the left ventricle in diabetic rats with *N*-acetyl cysteine, NAC treatment for 4 weeks. Furthermore, we clearly established that these defects in diabetic cardiomyocytes could be attributed to anomalous RyR2 behavior, as revealed by the spatiotemporal properties of both Zn^2+^ and Ca^2+^ sparks that especially exhibited slower kinetics and higher frequencies. These alterations can coincide perfectly with the behaviour of leaky RyR2 under strong oxidative/nitrosative stress. Indeed, it is known that both acute and chronic hyperglycemia trigger several biochemical and electrophysiological changes resulting in impaired cardiac contractile function [2], at most, due to hypoglycemia-induced big amount of reactive oxygen species, ROS, and followed by tissue/cell damage in several target organs including heart [3–5, 43]. Furthermore, it has been demonstrated that RyR2s are modulated with sulfhydryl oxidation in cardiomyocytes biphasically [25] while high glucose attenuates protein S-nitrosylation via superoxide production [26], and nitric oxide (NO) mediates intracytoplasmic and intranuclear Zn^2+^ release [27]. Moreover, an important contribution of redox modification of RyR2 with direct and/or indirect action of oxidants into SR Ca^2+^ leak in cardiomyocytes has also been shown under various diseased heart models induced in animals [21–24]. Another supporting fact of our current hypothesis arises from the present data on rapidly increased resting free Zn^2+^ level in cardiomyocytes due to Zn^2+^ release from intracellular stores by reactive, ROS/RNS [11]. Moreover, ROS/RNS have been proposed to contribute to direct and/or indirect damage to cardiomyocytes in diabetes [28, 44], providing a close relationship between both increased and deleterious effects of intracellular basal free Zn^2+^ level in the heart. A group of supporting data has been previously shown by our group, in which we demonstrated that inhibiting SR-Ca^2+^ release or increasing Ca^2+^ load in a low Na^+^ solution suppressed or increased Zn^2+^ movements, respectively. Furthermore, we also showed that mitochondrial inhibitors significantly reduced Zn^2+^ transients as well as Zn^2+^ sparks. In addition, either oxidation by H_2_O_2_ or changing to acidic pH inhibited the Ca^2+^-dependent Zn^2+^ release. Taken into consideration the previous ones with the present data, we proposed that Zn^2+^ release results, in part, from Zn^2+^ displacement by Ca^2+^ ions from metalloproteins binding sites whose availability depends on pH and redox status of cardiomyocytes, or rather that Ca^2+^ triggers ROS production inducing changes in metal binding properties of metallothioneins and other redox-active proteins as well as more Zn^2+^ release from SR due to defective RyR2 [11, 12, 15, 28, 45]. Additionally supporting this hypothesis, we have also demonstrated that an increase in Zn^2+^ alters several enzymatic activities and leads to RyR2 phosphorylation as seen in pathological conditions. Moreover, another supporting data to this study has been given by Kamalov et al. [46]. Their data showed that the basal intracellular free Zn^2+^ level is increased by 70% in cardiomyocytes from male diabetic rats being parallel to an unbalanced oxidant-status/antioxidant capacity in the same heart preparations, and by over 200% in aldosteronism. Coordinated changes in the basal intracellular free levels of both Ca^2+^ and Zn^2+^ have recently been also reported by our group under experimental conditions (intracellular Zn^2+^ overload or intracellular Ca^2+^ decrease), as well as during a single beat (transients or elementary events) in the cells [12].

Taken together, these data suggest that one of the beneficial effects of NAC treatment, under *in vivo* or *in vitro* conditions, may improve cardiac muscle function by reversing a maladaptive defect in Zn^2+^ signaling besides already known Ca^2+^ signaling, being parallel or individual, in cardiac myocytes of diabetic rats.

### 4.2. Increased Intracellular Free Zn^2+^ Phosphorylates Intracellular Ca^2+^ Signaling Kinases and Transcription Factor NF-*κ*B

Notably, in here, we have shown that exogenously applied Zn^2+^ caused marked phosphorylation in RyR2 while there was no effect on the protein levels of both RyR2 and an accessory protein of RyR2 macromolecular complex, FKBP12.6, as well as higher phosphorphorylations in both PKA and CaMKII in a concentration-dependent manner, similar to hyperglycemia. These data are further supported, in part, with the fact that a Zn^2+^-binding protein calsequestrin resides in the SR and can bind up to 200 mol Zn^2+^ per mol, independently of binding up to 50 mol Ca^2+^ per mol calsequestrin. It is known as an association of Zn^2+^ with over 300 enzymes, where it can interact strongly with electronegative sulfur, nitrogen, and oxygen moieties in multiple coordination forms, serving catalytic and structural roles in maintaining active peptide conformations [47]. In addition to metalloenzymes, Zn^2+^ is most known for its ability to bind and stabilize proteins involved in gene regulation in domains called Zn^2+^-fingers, Zn^2+^-clusters, and Zn^2+^-twists [48]. Therefore, it is well accepted that intracellular free Zn^2+^ plays critical roles in the redox signaling pathway and maintains the normal structure and physiology of various cell types while it can be toxic to cardiomyocytes [11, 49, 50]. Although Zn^2+^ is a vital element for mammalian in a certain range, some triggers such as increased ROS/RNS, ischemia, and infarction lead to release of Zn^2+^ from proteins and cause myocardial damage [11, 51–54].

Therefore, it can be hypothesized that Zn^2+^ may compete with or substitute for metal ions crucial for the activity of signaling proteins. In addition to this hypothesis, it has been shown that Zn^2+^ has multiple functional effects on Ca^2+^/calmodulin-dependent protein kinase II (CaMKII) and modulates RyR1 binding to sarcoplasmic reticulum vesicles in skeletal muscle biphasically [55, 56]. Accordingly, it can be clearly seen that intracellular free Zn^2+^ signaling can easily interfere with that of Ca^2+^ signaling in cardiomyocytes, particularly under pathological conditions, underlying, in part, cardiac dysfunction. Moreover, supporting this last statement, Zn^2+^ is known to induce CaMKII autophosphorylation, inhibit protein tyrosine phosphatases [55–57] and voltage-gated Ca^2+^ channels in mammalian cells [58], and to be highly cytotoxic [59] while divalent metal ions influence catalysis and active-site accessibility in the cAMP-dependent protein kinase [60]. In here, although a likely role for such intracellular Zn^2+^ signals is the modulation of protein phosphorylation, it is a strong possibility that both activated/phosphorylated CaMKII and PKA due to increased intracellular free Zn^2+^ turn into a hyperphosphorylated RyR2 under high extracellular Zn^2+^ exposure. Therefore, increased intracellular free Zn^2+^, most probably a contribution of increased intracellular free Ca^2+^, under increased oxidative stress together with depressed antioxidant-defence in the cells via hyperglycemia induces important defects in excitation-contraction coupling of cardiomyocytes. This statement is further supported with our previous observation related to coordinated changes in the basal intracellular levels of both free Ca^2+^ and Zn^2+^ under experimental conditions, as well as during a single beat in isolated cardiomyocytes [12].

Thus, a major physiological role for Zn^2+^ may be the modulation of cell signaling cascades, especially those involving protein phosphorylation, and considering also that intracellular Zn^2+^ can rise quickly and can influence Ca^2+^ regulation [49, 58], the relative very low abundance of Zn^2+^ in cardiomyocytes suggests its availability as second messengers for gene expression [49, 61]. In an early study, Atar et al. [49] showed that external Zn^2+^ could activate gene expression in GH3 cells due to modification of expression of Zn^2+^ regulating proteins by changes in intracellular free Zn^2+^ cellular systems [62]. In addition, with later findings on a direct regulation of Zn^2+^-fingers of Zn^2+^-finger transcription factors by Zn^2+^ availability [61, 63], it can be accepted that intracellular free Zn^2+^ may serve as a second messenger for transcription factor activation, and therefore, gene expression in response to stress. Accordingly, our present data showed that intracellular free Zn^2+^ can directly and markedly phosphorylate a transcription factor NF-*κ*B in cardiomyocytes, of note much more range in hyperglycemia cells. Of particular interest in cardiomyocytes, our data suggest that Zn^2+^ or Zn^2+^ carrying metalloproteins may be released from internal stores during increased oxidative/nitrosative stress and thereby affect transcription/translation pathways [64].

Taken into consideration all present and also previously published data, in here, we report that an enhancement of antioxidant defence in diabetics, directly targeting heart, seems to prevent diastolic dysfunction, being associated with normalization of RyR2 macromolecular-complex, and thereby prevention of both Zn^2+^ and Ca^2+^ leaks leading to normalization of basal levels of intracellular free Ca^2+^ and Zn^2+^ in the heart. This is nicely in line with an early report performed on a rodent heart with hereditary muscular dystrophy, demonstrating accompany of intracellular Ca^2+^ overloading and oxidative stress with increased intracellular free Zn^2+^ [52]. Besides, Kamalov et al. [46] suggested that an optimal range of intracellular free Zn^2+^/Ca^2+^ ratio in cardiomyocytes and mitochondria must be preserved to combat oxidative stress. Thus, the increased cytosolic and mitochondrial free Zn^2+^ level are coupled to the induction of oxidative stress, while antioxidant effects result from the rise in cytosolic and mitochondrial free Zn^2+^ levels accompanied by a simultaneous activation of metal response element transcription factor-1 and its induction of such antioxidants as metallothionein-1 and glutathione peroxidase. However, with a long-term treatment, by maintaining both a cellular redox-status and intracellular Zn^2+^ level near to control level, antioxidant NAC prevents the subsequent alterations in intracellular Ca^2+^ homeostasis including both Zn^2+^/Ca^2+^ releases by RyR2, which leads to major defects in cardiac activity.
